# Differential Response of Tomato Plants to the Application of Three *Trichoderma* Species When Evaluating the Control of *Pseudomonas syringae* Populations

**DOI:** 10.3390/plants9050626

**Published:** 2020-05-14

**Authors:** María E. Morán-Diez, Eduardo Tranque, Wagner Bettiol, Enrique Monte, Rosa Hermosa

**Affiliations:** 1 Spanish-Portuguese Institute for Agricultural Research (CIALE), Department of Microbiology and Genetics, University of Salamanca, Campus de Villamayor, C/Duero, 12, 37185 Salamanca, Spain; eduardotranque@hotmail.com (E.T.); emv@usal.es (E.M.); rhp@usal.es (R.H.); 2 Department of Microbial and Plant Biotechnology, Centro de Investigaciones Biológicas “Margarita Salas” (CSIC), C/Ramiro de Maeztu, 9, 28040 Madrid, Spain; 3 Embrapa Environment, CP 69, Jaguariúna 13918-110, SP, Brazil; wagner.bettiol@embrapa.br

**Keywords:** *T. asperellum*, *T. harzianum*, *T. parareesei*, (hemi)biotrophic bacteria, systemic defense, biocontrol

## Abstract

*Trichoderma* species are well known biocontrol agents that are able to induce responses in the host plants against an array of abiotic and biotic stresses. Here, we investigate, when applied to tomato seeds, the potential of *Trichoderma* strains belonging to three different species, *T. parareesei* T6, *T. asperellum* T25, and *T. harzianum* T34, to control the fully pathogenic strain *Pseudomonas syringae* pv. *tomato* (*Pst*) DC3000, able to produce the coronatine (COR) toxin, and the COR-deficient strain *Pst* DC3118 in tomato plants, and the molecular mechanisms by which the plant can modulate its systemic defense. Four-week old tomato plants, seed-inoculated, or not, with a *Trichoderma* strain, were infected, or not, with a *Pst* strain, and the changes in the expression of nine marker genes representative of salicylic acid (SA) (*ICS1* and *PAL5*) and jasmonic acid (JA) (*TomLoxC*) biosynthesis, SA- (*PR1b1*), JA- (*PINII* and *MYC2*) and JA/Ethylene (ET)-dependent (*ERF-A2*) defense pathways, as well as the abscisic acid (ABA)-responsive gene *AREB2* and the respiratory burst oxidase gene *LERBOH1*, were analyzed at 72 hours post-inoculation (hpi) with the bacteria. The significant increase obtained for bacterial population sizes in the leaves, disease index, and the upregulation of tomato genes related to SA, JA, ET and ABA in plants inoculated with *Pst* DC3000 compared with those obtained with *Pst* DC3118, confirmed the COR role as a virulence factor, and showed that both *Pst* and COR synergistically activate the JA- and SA-signaling defense responses, at least at 72 hpi. The three *Trichoderma* strains tested reduced the DC3118 levels to different extents and were able to control disease symptoms at the same rate. However, a minor protection (9.4%) against DC3000 was only achieved with *T. asperellum* T25. The gene deregulation detected in *Trichoderma*-treated plus *Pst*-inoculated tomato plants illustrates the complex system of a phytohormone-mediated signaling network that is affected by the pathogen and *Trichoderma* applications but also by their interaction. The expression changes for all nine genes analyzed, excepting *LERBOH1*, as well as the bacterial populations in the leaves were significantly affected by the interaction. Our results show that *Trichoderma* spp. are not adequate to control the disease caused by fully pathogenic *Pst* strains in tomato plants.

## 1. Introduction

The genus *Trichoderma* includes strains used as biological control agents in agriculture. Although such capacity has mainly been described against plant pathogenic fungi and oomycetes [1,2], it has also been achieved against bacteria, virus, insects, and nematodes [3,4,5,6]. The biocontrol direct mechanisms of *Trichoderma* are traditionally based on competition for space and nutrients, and on antibiosis and parasitism by the production of hydrolytic enzymes and/or metabolites [7,8,9]. Indirect biocontrol by the induction of systemic defense in the host plant [10,11], as a result of the efficient colonization of the rhizosphere by *Trichoderma* [12,13], has been also reported. 

Plants have developed adaptive mechanisms to avoid or reduce the possible damage caused by pathogen attacks, and certain phytohormone-regulated mechanisms play key roles in enhancing the plant basal resistance after the detection of a pathogen. Traditionally, contacting with pathogen and non-pathogen organisms triggers a phytohormone networking leading to defense responses known as systemic acquired resistance (SAR) and induced systemic resistance (ISR), respectively [14]. SAR is commonly activated by local pathogen attack and is associated with the accumulation of salicylic acid (SA) and pathogenesis-related proteins (PR) [15]. SA-related defense is boosted against biotrophic or hemibiotrophic pathogens, such as *Pseudomonas syringae*, whereas ISR requires the accumulation of jasmonic acid (JA) and ethylene (ET) and is activated when the plant interacts with non-pathogenic rhizosphere fungi and bacteria [14]. JA and ET signaling are singly or coordinately essential for immunity to necrotrophs [16]. Overall, the phytohormone profile, including composition, quantities, and timing, varies among plants and depends greatly on the lifestyle and infection strategy of the invading organisms [17].

*Pseudomonas syringae* bacteria produce widely varying plant disease symptoms comprising blights, leaf spots, and galls. Strains of this pathogen use different strategies to break down the health of susceptible host plants [18], among which are survival and/or multiplication on the plant surface (epiphytic phase), the ability to enter into the apoplast through natural openings, such as stomata and wounds, the suppression of host immunity, and the establishment of an aqueous apoplast to facilitate the procurement of nutrients and water. Structural components of *P. syringae* bacterium (i.e., flagellin or peptidoglycan) are recognized by host cell transmembrane proteins known as pattern recognition receptors (PRRs), triggering the induction of plant immune responses. To circumvent such responses, *P. syringae* has acquired the *hrp* (hypersensitive response and pathogenicity) gene cluster—for the regulation and biosynthesis of a syringe-like structure known as type III secretion system (T3SS) and a core type III effector genes repertoire—together with the production of a significant array of phytotoxins (i.e., coronatine, syringomycin, phaseolotoxin, etc.) and other virulence factors, all of which are crucial for altering plant metabolism and physiology to promote infection by different *P. syringae* pathovars [18]. 

In particular, the *P. syringae* pv. *tomato* strain DC3000 (*Pst* DC3000) is able to synthesize the polyketide coronatine (COR), a non-host-specific toxin produced by strains belonging to at least five pathovars of this bacterial species [19]. COR is structurally very similar to JA-isoleucine (JA-Ile) and both share the same coreceptor complex, CORONATINE INSENSITIVE1 (COI1)-JASMONATE ZIM domain (JAZ), in plants to regulate responses to JA signaling [20]. Indeed, COR is more active than JA-Ile in triggering the COI1–JAZ interaction in vitro, resulting in the proteasome-mediated degradation of JAZ proteins that suppress positive regulators of the JA-dependent defense, such as *MYC2* [21]. This means that the JA signaling pathway becomes derepressed, initiating the expression of a large number of JA-responsive genes. In this way, COR can lead to leaf chlorosis, anthocyanin production, ethylene (ET) emission, indole-acetic acid (IAA) synthesis, the inhibition of stomata closure and the rapid induction of its opening, bacterial growth in the apoplast, systemic susceptibility, and disease symptoms [22]. Since COR mimics JA-Ile, it is used by *Pst* DC3000 to suppress stomatal defense and SA-mediated plant defenses through SA–JA antagonistic crosstalk [23]. This strain also rapidly induces abscisic acid (ABA) biosynthesis in *Arabidopsis* to antagonize SA accumulation [24]. However, other studies have shown that jasmonates synergize SA defenses [25,26] and that JA dynamics during *Pst* DC3000 infection of *Arabidopsis* is inconsistent with a scenario of JA antagonizing SA signaling, in which JA was accumulated very late in the infected leaf, whereas regulatory proteins such as JAZ5 and JAZ10 were involved in reducing COR toxicity [27]. In addition, the production of IAA and an enzyme involved in ET biosynthesis may contribute to the overall virulence of *Pst* DC3000 [28]. 

*Trichoderma* activate systemic resistance through multiple hormonal signaling pathways [29]. This *Trichoderma* ability was firstly described for *T. asperellum* in cucumber as JA/ET-dependent ISR [30], although, in a later study on *T. harzianum*–*Arabidopsis* interaction, it was detected that the SA-independent ISR also required ABA in addition to JA/ET [31]. Likewise, *Trichoderma* concertedly increases JA/ET and auxin responses in the plant to overcome SA-dependent defenses [32]. However, experiments on *T. asperellum* and cucumber also demonstrated that the fungal strain, interaction time, and inoculum concentration determine the changes in SA and JA levels [33]. Several studies performed with different plant species and *Trichoderma* strains have shown that the SA pathway is also key for a *Trichoderma*-boosted ISR response, and the simultaneous induction of genetic markers from SAR and ISR pathways has been detected in plants challenged by *Trichoderma* [13,34,35,36,37]. In addition to the whole array of proteins and metabolites of *Trichoderma* associated with the modulation of plant defense and development responses [26], these fungi also produce the phytohormones IAA [38] and SA [39], detoxifying enzymes for reactive oxygen species (ROS) scavenging [40] and regulate the endogenous ET levels in the plant [41], all of which affect plant responses. Recent studies even indicate that *Trichoderma* reprograms the plant defense by adapting the SA- and JA-dependent defenses according to the pathogen infection stage [5,42].

Few studies have used bacterial strains as a target to analyze the biocontrol potential of *Trichoderma*. It has been reported that *T. asperelloides* (formerly *T. asperellum*) T203 triggers systemic resistance to *P. syringae* pv. *lachrymans* in cucumber [43] and to *Pst* DC3000 in *Arabidopsis* [44]. Tomato plants inoculated with *T. atroviride* and *T. virens* also displayed systemic protection ability against *Pst* DC3000 [6], although the infection by pathogens on *Trichoderma* pre-treated plants generated more complex outcomes than those expected [6]. To address the possibility that the priming of defense responses against *Pst* on tomato plants may be a common ability associated with *Trichoderma* species, we analyzed the effects of three different *Trichoderma* strains, *T. parareesei* T6, *T. asperellum* T25, and *T. harzianum* T34, to induce systemic defense against two different strains of the foliar pathogen *Pst*, DC3000 and DC3118, which differ in COR production and in virulence to tomato plants. In this study, *Trichoderma* was applied on the seeds, *Pst* infection was performed by dipping application to the leaves in four-week-old plants, and the measurements of the expression of defense-related genes and *Pst* levels were made 72 h post-inoculation (hpi) with the foliar pathogen. Our results show that, after a challenge with DC3000 or DC3118, different gene expression profiles were detected in plants from the three *Trichoderma* treatments. Whereas protective effects of all *Trichoderma* strains against DC3118 were observed, in terms of *Pst* population levels, only a minor protection was achieved by *T. asperellum* T25 in tomato plants infested with the fully pathogenic strain DC3000.

## 2. Results and Discussion

### 2.1. Bacterial Population Sizes in Tomato Plants Are Defined by Trichoderma Species Specificity 

**Figure 1:**
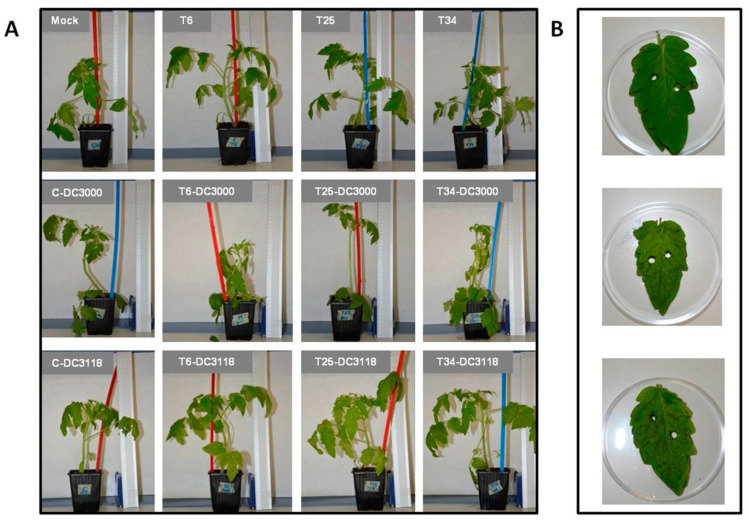
Experimental setup of tomato plants and their interaction with *Trichoderma* strains and *P. syringae* strains. (**A**) Four-week-old plants dip-inoculated with *Pst* strains at 72 hpi (DC3000, center row and DC3118, bottom row) and control plants (top row). (**B**) Disease symptoms on a pool of 8 leaflets used for counting bacterial populations (Mock, top; C-DC3000, center; and C-DC3118, bottom).

In order to evaluate whether the *Pst* population size of pathogenic (DC3000) and COR-deficient (DC3118) strains were determined by the effect of three different *Trichoderma* treatments (T6, T25, and T34) on tomato plants, colony-forming units (CFU)/cm^2^ leaf values were recorded for each treatment (Table 1). Control plants not challenged with *Pst* (Mock, T6, T25, and T34) did not show any disease symptoms (Figure 1, top row in A and top in B) or bacterial growth on the leaf surface. The dry biomass and plant height did not show statistical differences among treatments (Table 2). Similar results have been reported by other studies that indicate the absence of significant differences in stem length, height, or dry biomass in 4-week-old tomato plants treated with *T. parareesei* T6 or *T. harzianum* T34 [45,46]. The effect of some *Trichoderma* species on plant growth enhancement is well documented [6,9,10,37]; however, an increased number of research studies are suggesting that this effect appears not to be a ubiquitous trait in all *Trichoderma* species but being strongly connected and driven by different factors, such as the host-species specificity, the application method used, as well as the environmental growth conditions considered for the study [47,48,49,50,51]. 

When considering *Pst* in the plant–microbe interaction systems, plants inoculated with DC3000 displayed a higher severity of the disease than those inoculated with DC3118, with the detached branches standing out from the first and second set of true leaves in all treatments (C-DC3000, T6-DC3000, T25-DC3000, and T34-DC3000) (Figure 1A) as well as a higher disease severity index (Table 1). This symptomatology would be in agreement with the limited virulence displayed by the *Pst* DC3118 strain in *Arabidopsis* plants [52,53,54]. Although DC3118-inoculated plants showed similar necrotic lesions to those observed in plants inoculated with DC3000 (Figure 1B), the bacterial population sizes were higher in those plants inoculated with DC3000 (Table 1, first row). These results are likely explained by the fact that other factors seem to contribute to virulence in *Pst* strains, such as the syringomycin phytotoxin or the number of T3SS effectors secreted by this species [18,55]. Uppalapati et al. [56] observed no differences between bacterial populations of COR-deficient and pathogenic *Pst* strains on four-week-old tomato plants at 1 and 3 days post-inoculation (dpi), though differences were significant at 6 dpi. However, these authors also observed different disease lesion phenotypes on DC3000-treated plants and those plants inoculated with DC3118 at 6 dpi. We should not forget that, as occurs in *Trichoderma* [33], other factors such as experimental conditions and interaction times can also play an important role. 

Bacterial populations of the strain DC3118 were significantly reduced by *Trichoderma* strains (Table 1). *Trichoderma parareesei* T6, *T. asperellum* T25, and *T. harzianum* T34 reduced the DC3118 growth in leaves by 64.7%, 63.7%, and 17.3%, respectively, in relation to the control (Table 1). However, minor control was achieved by *T. asperellum* T25 against DC3000 because the counts of this bacterium in tomato leaves were only reduced by 9.4%. For the three *Trichoderma* strains used, better protection was obtained against the less pathogenic bacteria. In an *Arabidopsis*-*Pst*-*Trichoderma* system, Brotman et al. [44] observed that plants pre-treated with *T. asperelloides* displayed reduced bacterial population in both hydroponic and soil systems 48 h before being challenged with DC3000. Our results show that, despite the ability of the three *Trichoderma* species to biocontrol DC3118 populations, only T25 displayed significant differences when testing the pathogenic *Pst* DC3000 strain. Different results probably could have been obtained without a surfactant-facilitated infection protocol and if it had been performed at a different time point. However, the COR-deficient strain was more effectively blocked by the three *Trichoderma* strains, even in the presence of the surfactant, and a much better control rate could have been obtained without the assistance of this compound in the bacterial entering. Given that COR plays a key role in the virulence exhibited by this pathogen [18,55], the linkage between the *Trichoderma* species specificity and the plant responses seems evident, due not only to *Trichoderma* itself but also to the specific pathogenic strain attacking the plant host. This specificity has been described by other authors, whose studies indicate that the *Trichoderma* species´ behavior against different pathogens in tomato plants is clearly shaped by an existence of species specificity [6,37]. 

### 2.2. Tomato Plants Challenged with Pst Strains Proved to Be Highly Synergistic in SA and JA Signaling Defense Pathways

**Figure 2:**
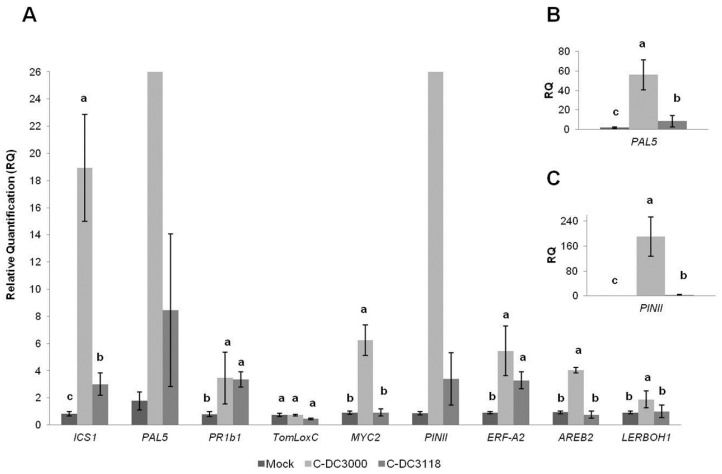
Relative expression of defense-response genes in 4-week-old tomato plants inoculated with *P. syringae (Pst*). (**A**) The samples were collected 72 h after mock-inoculated plants (Mock, control plants untreated neither with *Trichoderma* nor with *Pst*) or *Pst*-inoculated plants (C-DC3000 and C-DC3118) using an MgCl_2_-surfactant solution for 10 s. Four biological replicates per treatment with three technical replicates each were used in the analysis. The data are displayed as the relative quantity (RQ, 2^−∆∆Ct^) + standard deviations of target genes compared to those of their basal condition (Mock). The expression values of each gene were normalized to the quantity of the actin gene used as the endogenous gene. (**B**,**C**) Plot detail with the relative expression of *PAL5* and *PINII* genes, as shown in panel (**A**). The values in each bar followed by a different letter differ significantly (Tukey’s test at *p <* 0.05) when compared among treatments (Mock, C-DC3000, and C-DC3118) within each gene.

It is well known that plant activates SA-dependent defenses as a protective response against *Pst*, while the pathogen tries to suppress such a defense by the JA-mimicking activity of COR [54,56,57]. Among all genes tested in our study (Table S1), *ICS1*, *PAL5*, *PR1b1*, *PINII*, and *ERF-A2* genes showed significant differences compared to Mock plants (Figure 2 and Table S2) under the *Pst* DC3118 treatment, while plants dip-inoculated with DC3000 showed significant upregulation of all genes tested with the exception of *TomLoxC* when comparing with Mock plants, at least at 72 hpi. The absence of COR toxin production in DC3118 could be related to the absence of the upregulation of the JA-responsive defense gene *MYC2* and the ABA-dependent defense gene *AREB2*. In any case, plants inoculated with DC3118 showed a higher expression of the SA-defense genes tested, *PR1b1* (SA-triggered pathogenesis-related protein), *ICS1* (biosynthesis of SA by the isochorismate pathway), and *PAL5* (involved in the conversion of _L_-phenylalanine into *trans*-cinnamate, the initial committed step of the multi-branched phenylpropanoid biosynthetic pathway for SA and lignin synthesis), than Mock plants. These results are consistent with the activity of SA defense upon *Pst* attack, independent of COR production. *PR1b1* did not show in this case significant differences when compared between *Pst* strains treatments. The strong upregulation of *PAL5*, notably DC3000 treatment, could be indicative of a triggering of lignin synthesis, which should help to prevent the spread of the pathogen by spatial restriction. In fact, lignin synthesis and deposition are known to occur upon infection by pathogenic bacteria [58,59]. Regarding, SA-responsive defense marker genes, a different outcome was observed by other authors in tomato plants challenged with *Pst* strains and evaluated at 24 hpi, where a suppression of SA-mediated defense by COR has been suggested [56]. In a report of a study performed at a later time point, contrary to what we have observed, *PR1b1* downregulation was detected in plants inoculated with DC3000 compared to the expression in plants treated with DC3118 [56]. These results would be confirming that the tomato plant needs to activate an SA-dependent defense against *Pst* at an early stage somehow, and that the onset time of the SA and JA signaling pathways is clearly essential for SA–JA antagonistic crosstalk and the final output of the gene expression after a given interaction time [57]. 

Compared to SA-responsive genes, a very strong upregulation of the JA-dependent marker gene *PINII* was observed in DC3000-treated plants (Figure 2C), which is compatible with the mode of action of COR. The induction of JA-mediated defense in tomato plants leads to the accumulation of several compounds, including proteinase inhibitors (PINs) whose synthesis involves the expression of *PINII* [60]. These authors observed a predominant expression of *PINII* in DC3000-inoculated plants at 7 dpi, with no significant differences for *PR1b1* expression when compared to Mock plants. Similarly, *PR1b1* expression (Fold change (FC) 3.47 + 1.91) was significantly reduced in comparison to *PINII* in DC3000-treated plants (FC 190.73 + 62.55) (Figure 2A,C, respectively; Table S2) as well as when compared to the SA-biosynthesis *PAL5* gene (FC 56.09 + 15.41) (Figure 2 and Table S2). This could be explained by a more prolonged defense priming effect modulated by JA at the expense of a reduced and more transient SA-dependent defense. In the DC3000-treated plants of our study, *PR1b1*, *ISC1*, and *PAL5* expressions were significantly lower in comparison to *PINII*, with a far higher relative expression ratio (FC 190.73 + 62.55), which is consistent with a COR effect lasting until at least the third dpi. There might be a key role of COR in the increased expression levels detected in DC3000 for *ICS1*, *PAL5,* and *PINII* genes as well as how the balance between SA and JA/ET might affect the responses against this strain [18,57,61]. The significant upregulation of *AREB-A2* in plants inoculated with DC3000 is in agreement with the previously described activation of ABA production in response to this same strain [24]. However, the expected antagonism between SA and ABA was not detected in our study since the upregulation of genes involved in SA signaling did not block the ABA-dependent defense pathway, rather the contrary in response to the fully pathogenic *Pst* strain. Moreover, the increased *PAL5* expression detected in plants challenged with DC3000 was not accompanied by an expected increase in the phenylalanine ammonia-lyase (PAL) activity (Table 3). This observation is consistent with a scenario where limited PAL enzymatic activity leads to a reduction in the phenylpropanoid biosynthesis of SA and thus the ABA accumulation required for *AREB2* upregulation, at least at 72 hpi. However, SA can be also produced by an alternative pathway, as confirmed by the increased expression of both the *ICS1* and *PR1b1* genes in plants inoculated with both *Pst* strains.

Other JA- and JA/ET-mediated defense genes that showed a significant upregulated expression in plants challenged with DC3000 compared to Mock and DC3118, and Mock plants, respectively, were *MYC2* and *ERF-A2* both encoding transcription factors (Figure 2A). *MYC2* and *ERF1* branches of the JA signaling pathway are mainly activated by wounds/insects but also by pathogen attack, including *P. syringae* [61]. Additionally, *MYC2* seems to be involved in the repression of *ICS1* through the NAC transcription factor family in *Arabidopsis* plants [62]. As might be expected, the upregulation of these two JA-signaling-related genes matched with the star of the upregulation of the SA biosynthesis *ICS1* gene. The increased expression of *ERF-A2* is in agreement with the previously described activation of ET production by DC3000 [28]. Although it could be expected that an interplay between SA and JA optimizes the immune response against *P. syringae* [17], our results show that, at least at 72 hpi, both SA- and JA-signaling defense pathways are activated in tomato plants in response to DC3118 and DC3000, the latter to a larger extent. This points in the same direction as other previous studies in which JA and SA defenses act synergistically to reduce COR toxicity [25,26,27].

The consistent upregulation of SA- and JA-responsive genes in tomato plants inoculated with DC3000, this being indicative of the regulation of the JA-responsive defense response by the COR produced by this strain, is in agreement with the higher disease index achieved with DC3000 and its higher population sizes recorded on 4-week-old tomato leaves at 72 hpi (Table 1). 

### 2.3. Tomato Plants Challenged with Pst Strains under Different Trichoderma Treatments Display A Complex Defense-Related Gene and Plant Hormone Signaling Networks

**Figure 3:**
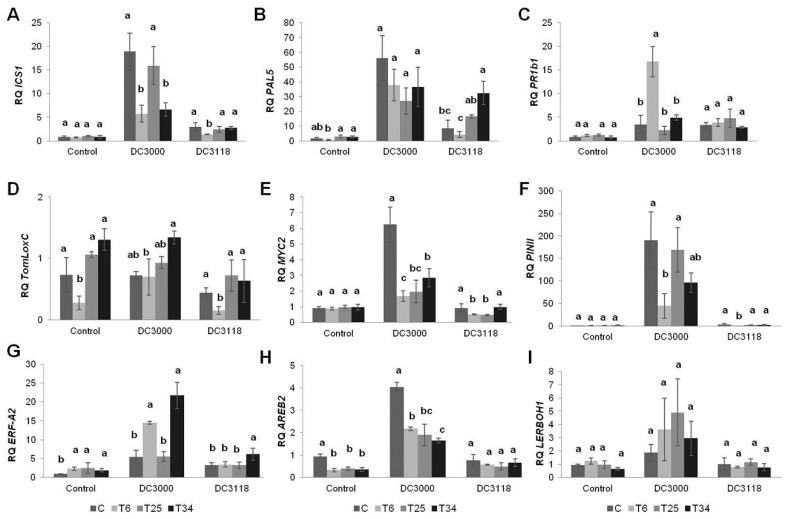
Relative expression of defense-response genes in 4-week-old tomato plants dip-inoculated with *P. syringae* strains (DC3000 and DC3118) or without *Pst* (Control) and treated with *Trichoderma* strains (T6, T25, and T34). Samples were collected at 72 hpi of *Pst* strains. Four biological replicates per treatment with three technical replicates each were used in the analysis. The data are displayed as the relative quantity (RQ, 2^−∆∆Ct^) + standard deviations of target genes compared to those of their basal condition (Mock (C), control plants untreated neither with *Trichoderma* nor with *Pst*). The expression values of each gene were normalized to the quantity of actin gene used as the endogenous gene. The values in each bar followed by the same letter do not differ significantly (Tukey’s test at *p <* 0.05), for Control-, DC3000-, and DC3118-treated plants, respectively. Panels A–I correspond to the relative expression of genes: *ICS1* (**A**), *PAL5* (**B**), *PR1b1* (**C**), *TomLoxC* (**D**), *MYC2* (**E**), *PINII* (**F**), *ERF-A2* (**G**), *AREB2* (**H**), and *LERBOH1* (**I**).

We investigated whether the different effects observed on bacterial population sizes in plants inoculated with *Trichoderma* were correlated to a specific activation of defense-related genes (Table S1) to explain the differential biocontrol patterns observed among strains. Control plants treated with *Trichoderma* but not challenged with *Pst* strains presented no significant differences among most of the sets of genes evaluated in this study when compared with their control (Mock) (Figure 3). Exceptions were identified for genes *TomLoxC*, *ERF-A2*, and *AREB2* (Tukey´s test, *p <* 0.05) with different outcomes based on the *Trichoderma* strain applied. When compared to the control, tomato plants treated with T6 displayed the upregulation of *ERF-A2* in a similar way to what is observed with T25 and T34. T6 also displayed the downregulation of *TomLoxC* and *AREB2,* the latter also being downregulated in response to the other two *Trichoderma* strains (Figure 3). Similarly, a marked *AREB2* downregulation was observed in tomato plants challenged with *T. harzianum* under saline stress beside the non-inoculated control [46]. The undulating mutual antagonistic effect described for ET- and ABA-signaling defense genes in T6–tomato interaction [45] is compatible with the expression behavior of *ERF-A2* and *AREB2* observed for the three *Trichoderma* strains. Tucci et al. [37] explained that some plant varieties as well as the different responses triggered by *Trichoderma* strains displayed a more varied response on defense-related genes than others. T6-treated plants in the absence of *Pst* might be a good example. Interestingly, both T25 and T34 appeared to modify phytohormone-signaling defense pathways, operating in a similar way. However, they showed a different ability to reduce the levels of DC3118 (Table 1). In this particular case, *MYC2* and *ERF-A2* were upregulated in plants treated with T34 in comparison to T25, which would indicate that JA/ET-signaling pathways do not play a key role in the defense against this strain. It has been reported that T6 displays an already described zigzag model of defense-related gene expression at short times (up to 6 dpi) in 4-week-old tomato plants [2,45]. Based on the slightly differential expression patterns we have observed, the different activation of defense-related genes among *Trichoderma* strains could be the cause of the later response we detect against *Pst* strains, preparing the plant for the attack. Is the presence of *Pst* strains, mainly DC3000, along with the *Trichoderma* strains the trigger for a differential activation of plant defense genes? Comparisons made among *Trichoderma* strains and against each individual *Pst* strain revealed a complex system of a signaling network of defense-related genes, as this would probably be expected for a three-component system with plants growing under simulated field conditions. 

To our knowledge, only one study has attempted to analyze the effect of *Trichoderma* species on the biocontrol of *Pst* in tomato plants [6]. Our approach aimed to contribute, through a comparative analysis, to the understanding of the molecular mechanisms used by *Trichoderma* to trigger immune responses in tomato plants when subjected to the action of pathogenic and COR-deficient strains of *Pst*. The only *Trichoderma* strain that proved to differently reduce the level of DC3000 in tomato plants was T25, which might be explained by the significant *MYC2* and *AREB2* downregulation (Figure 3). Only the *ERF-A2* and *AREB2* gene expression was significantly different in T25-treated tomato plants compared to the Mock (Figure 3). These results would indicate that T25 keeps similar levels of expression for the SA-dependent defense genes while increasing the levels of genes involved in ET-dependent defense, providing the expected conditions for the pathogenic bacteria to be controlled. 

The downregulation of *AREB2* in plants treated with any of the *Trichoderma* strains in the presence of *Pst* DC3000 (Figure 3) supports the fact that the activation of ABA signaling antagonizes the SA-dependent responses [63]. A different outcome was observed for *ERF-A2* expression that was upregulated in the T6-DC3000 and T34-DC3000 treatments. It has been shown that, though infection of *Arabidopsis* plants with DC3000 does not alter the expression of ET response factor genes, its overexpression increases the plant susceptibility to this pathogen [63,64]. Precisely, plants treated with T25 maintained similar *ERF-A2* expression to their DC3000-infected control (C-DC3000) (*p <* 0.05), such susceptibility not being enhanced in this case (Table 1). 

There are other genes that might contribute to the understanding of these behaviors. Considering the *PR1b1* upregulation in plants treated with T6 (T6-DC3000), we might hypothesize a similar correlation between SA- and JA-activated genes. However, in this particular case, it is unclear whether *PR1b1* plays a more significant role or not, and a mutual antagonistic effect with *PINII* could not be excluded. A different outcome was observed for plants inoculated with DC3118 and pre-treated with any of the three *Trichoderma* strains (Table 1). The lowest DC3118 population levels were obtained in plants treated with T6 and T25. Our findings suggest that SA-dependent defense genes should be upregulated, while those involved in JA signaling pathways might be down-regulated due to the lack of the COR toxin in DC3118. However, the results show that, in response to DC3118 and compared to C-DC3118, *PAL5* was only upregulated in T34-treated plants (Figure 3) that bear no relation with the control of this strain exerted by T6 (Table 1), clearly the only *Trichoderma* strain able to downregulate *PINII*. Interestingly, the minor protection achieved by the T34 strain against DC3118 was accompanied with a significant increase in *ERF-A2* expression, the significant activation of ET-signalized responses in the T34–DC3118 interaction could be associated with a negative effect to control this bacterial pathogen. As lower levels of *MYC2* were also detected in T6- and T25-treated plants than those in T34 or C-DC3118 plants (Figure 3), an activation of JA/ET-signaling defenses by the T6 and T25 strains could not be excluded. Though no significant differences (*p* < 0.05) were observed when evaluating PAL activity data referred to treatments with or without *Pst* strains (Table 3), higher PAL activity was detected in plants treated with T34 and infected with DC3118 compared to Mock and DC3000-treated plants. Therefore, it is difficult to provide, in three-player systems, a straightforward response, the primary defense responses to which deployed by tomato plants depend on *Pst* attack and are triggered by the application of *Trichoderma*.

**Figure 4:**
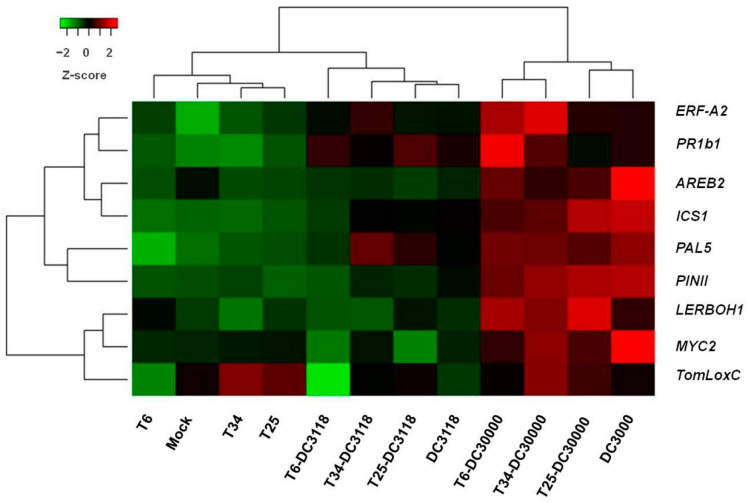
Two-way hierarchical clustering analysis of the normalized expression level of the nine genes evaluated in this study and the 12 treatments considered. Low gene expression values are green, and high gene expression values are red. The distance matrix was calculated using the Ward’s linkage method and the Euclidean distance, and R programming tools were used to visualize the matrix.

The complexity of working with an interconnected system of plant-pathogenic bacteria-biocontrol agent under in vivo conditions can surely help us to respond to many of our questions regarding these systems but also brings us to more unsolved questions, particularly those due to the fact that *Trichoderma’s* plant induced-responses have been described as a time-dependent undulating process [2,45]. Two-way clustering was used to summarize the grouping gene expression changes that behaved similarly across groups of treatments (Figure 4). As discussed above, we can identify here two separated clusters, one being assigned to those plants treated with DC3000 and the second one to those plants that are untreated, treated with the less-pathogenic *Pst* strain, or treated with a *Trichoderma* strain (Figure 4). It is worth noting here that the different behavior of the T6 strain is an independent branch within the *Pst* DC3118 cluster and the Mock-*Trichoderma* cluster. The behavior across genes mirrors the complexity of hormone signaling, discussed above, displaying a non-defined SA/JA-ET pattern within each cluster of genes (Figure 4). 

Despite this complex behavior in terms of signaling pathways, there is a clear effect of the variable “*Pst*” and variable “*Trichoderma*” on gene expression, bacterial population, and the severity of the disease. Moreover, there was a significant effect upon gene expression due to the interaction of both variables (Table S2, *p* < 0.05). Only *LERBOH1* expression changes did not appear to be significant due to the combination of *Pst* and *Trichoderma*; in this case, there were no differences due to any of the *Trichoderma* strains (Table S2). Similarly, bacterial population sizes were affected by *Pst* and *Trichoderma* strains but also by the combination of both (Tukey’s test, *p* < 0.05). There was, however, no significant effect of combining both organisms on the severity of the disease (Tukey’s test, *p >* 0.05). 

## 3. Materials and Methods 

### 3.1. Microorganisms and Tomato Seeds

Three *Trichoderma* strains, belonging to three different species, were obtained from culture collections and used in this study: *T. parareesei* (formerly *T. reesei*) IMI 113135 (CABI Bioscience, Egham, UK), *T. asperellum* IMI 296237 [65], and *T. harzianum* CECT 2413 (Spanish Type Culture Collection, Valencia, Spain). They are here referred to as the T6, T25, and T34 strains, respectively, and were grown and sporulated on potato dextrose agar (PDA, Difco) at 28 °C in the dark. 

The bacterial strains used in this study, kindly provided by Roberto Solano (CNB-CSIC, Madrid, Spain), were the phytopathogen *Pseudomonas syringae* pv. *tomato* DC3000 (rifampicin resistant) (*Pst* DC3000 or DC3000) and the COR-deficient *P. syringae* pv. *tomato* DC3118 mutant (rifampicin, kanamycin and spectinomycin resistant) (*Pst* DC3118 or DC3118). The bacterial strains were streaked onto Luria-Bertani (LB) agar plates supplemented with the suitable antibiotic and grown at 28 °C for 48 h for further use. 

Tomato seeds (*Solanum lycopersicum* “Marmande”) (Eurogarden, Barcelona, Spain) were surface disinfected in 70% ethanol for 10 min followed by an additional step of 10 min in a 2% sodium hypochlorite solution. Seeds were then rinsed with sterile water five times before their use. 

### 3.2. Experimental Design and Plant Treatments

*Trichoderma-*conidial suspensions were harvested from sporulated-PDA plates with 10 mL of sterile water and filtered through glass wool to remove mycelia. Conidia concentration was determined using a hemocytometer chamber. Forty surface-disinfected tomato seeds per treatment were coated with 1 mL of 10^8^ conidia/mL-suspension of each *Trichoderma* strain and air-dried in open Petri dishes under aseptic conditions in a laminar air-flow hood. Control seeds were dipped in 1 mL of sterile water and air-dried under similar conditions than treatments.

Seeds were sown in plastic pots (9 × 9 × 10 cm) containing a 3:1 mixture of substrate (50% clay to 50% cocopeat; 0.8 kg/m^3^ of NPK 14-16-18 (N-P_2_O_5_-K_2_O) and pH 6–6.5) and vermiculite. One plant per pot was grown in a greenhouse under controlled conditions of humidity (75%), a 16 h photoperiod, and a temperature of 18–28 °C.

For plant inoculation, a single bacterial colony was cultured in 5 mL of LB medium supplemented with the suitable antibiotic for each *Pst* strain at 28 °C via shaking for 12–16 h. This culture was used as a starter to inoculate 100 mL of LB-antibiotic medium that was incubated at 28 °C until a log phase was reached. Bacterial cells were collected by centrifugation at 2500× *g* for 5 min, washed with sterile water, and resuspended in 10 mM MgCl_2_ until an OD_600_ of 0.005 was reached (approximately 1 × 10^8^ colony-forming units (CFU)/mL). Bacterial suspension was supplemented with 0.03% of the surfactant Silwet L-77 (Sigma-Aldrich, Madrid, Spain) to increase bacterial leaf infiltration. Four-week-old tomato plants displaying a second set of true leaves were inoculated by dipping the whole rosette leaves into a sustained stirred-bacterial solution for 10 s. Plants were incubated in plastic domes to keep a high humidity percentage with a 16 h light photoperiod for 72 h.

Experimental setup was carried out with four tomato plants per treatment in a randomized design. Treatments were assigned as follows: Mock (untreated control plants); C-DC3000 and C-DC3118 (plants dip-inoculated with each strain of *Pst*); T6, T25, and T34 (plants treated with each *Trichoderma* strain), T6-DC3000, T25-DC3000, and T34-DC3000 (plants treated with each *Trichoderma* strain and DC3000-inoculated); T6-DC3118, T25-DC3118, and T34-DC3118 (plants treated with each *Trichoderma* strain and DC3118-inoculated). The MgCl_2_-surfactant solution was applied as a mock inoculation to control (Mock) and *Trichoderma*-treated plants (T6, T25, and T34) (Figure 1). 

For measuring bacterial population in tomato plants, four opposite leaflets of the stems on the second set of true leaves were detached per plant (Figure S1). Leaves were piled up to cut two sets of four discs (0.5 cm^2^) from each side of the leaf midrib by using a hole punch. A total of eight discs per plant were collected and placed in a microtube containing 1 mL of sterile water and serial dilutions of 10^−2^, 10^−3^, and 10^−4^ were used for bacterial quantification spreading 100 µL of each dilution onto King’s B (KB) agar medium plates supplemented with the suitable antibiotic for each *Pst* strain, with three technical replicates per dilution. Plates were incubated at 28 °C until colonies were developed and counted. For gene expression, four opposite leaflets on the stems of the second set of true leaves (Figure S1) were collected and frozen in liquid nitrogen for further RNA extractions. Additionally, disease severity was evaluated visually on the same set of plants and scored using a disease index with a range from 0 to 3 (0 = healthy-looking plant; 1 = 1 to 2 branches detached from the plant; 2 = 3 to 4 branches; 3 = more than 4) [66].

For evaluation of the effect of *Trichoderma* strains on the development of the plants, measurements of the stem were taken from the hypocotyls to the top of the youngest leaflet of each plant. After measuring, plants were oven-dried at 65 °C and weights were recorded. 

### 3.3. RNA Isolation, cDNA Synthesis and Real-Time Quantitative PCR (qRT-PCR)

A four-leaflet pool per plant was collected as a biological replicate for RNA extraction. Four biological replicates per treatment were considered for this study. The isolation of RNA was obtained from 100 mg of fresh tissue with TRIzol reagent (Invitrogen Life Technologies, Carlsbad, CA, USA) and treated with DNase RQ1 (Promega, Spain) for the synthesis of first strand cDNA using an oligo(dT) primer (Takara Inc., Tokyo, Japan), following the manufacturer’s protocol. Nine marker genes representative of SA (*ICS1* (isochorismate synthase protein 1) and *PAL5* (phenylalanine ammonia lyase protein 5)) and JA (*TomLoxC* (lipoxygenase protein C)) biosynthesis, SA- ((*PR1b1* (pathogenesis-related protein 1b1)), JA- (*PINII* (wound-induced proteinase inhibitor protein II), and *MYC2* (transcription factor MYC2)), and JA/ET-dependent (*ERF-A2* (ethylene response factor protein A2)) defense pathways, and *AREB2* and *LERBOH1* genes encoding an ABA-responsive element binding protein and an NADPH oxidase involved in oxidative burst, respectively, were analyzed in tomato plants (Table S1) by qRT-PCR using a SYBR FAST KAPA kit (Biosystems, Buenos Aires, Argentina) in a StepOnePlus thermocycler (Applied Biosystems, Foster City, CA, USA). These marker genes were selected because of their usefulness in previous transcriptomic studies on *Trichoderma*–tomato interactions [45], fungus [67], and nematode [5] attack. All reactions were performed in triplicate in a total volume of 10 µL. The primers used to check the expression of defense-related genes in tomato plants are listed in an additional file (see Table S1) with their references, slopes, and efficiencies calculated from standard curves of pooled cDNA samples (Applied Biosystems software). Relative expression levels were calculated from the threshold cycle (Ct) using the 2^−ΔΔCT^ method [68] and the α-actin transcript as an internal reference.

### 3.4. Determination of _L_-Phenylalanine Ammonia-Lyase (PAL) Activity

The enzymatic activity was analyzed on the same plant material used for RNA extractions for each treatment. Tissue was homogenized in 3 mL of 0.1 M trisodium borate buffer (pH 8.5) containing 1.4 mM 2-mercaptoethanol and 0.1 g of insoluble polyvinylpyrrolidone. The extract was filtered through cheesecloth, and centrifugation was carried out at full speed for 15 min. The determination of PAL activity was analyzed as the rate of conversion of _L_-phenylalanine into trans-cinnamic acid at 270 nm in a spectrophotometer. A 200 μL aliquot of the sample was added to 400 μL of borate buffer and 200 μL of 40 mM _L_-phenylalanine, and the reaction mixture was incubated at 37 °C for 15 min. The reaction was stopped with an equal volume of 10% (*w/v*) of trichloroacetic acid (TCA), and samples were centrifuged at full speed for 15 min. The supernatants were used to measure the absorbance at 270 nm, and trans-cinnamic acid was calculated using a standard curve according to Lee et al. [69]. The results are presented as μmol/min (U) per g of fresh tissue. 

### 3.5. Data Analysis

All data were collected from four biological replicates. The homogeneity of variances and normality tests were performed by Bartlett’s and Shapiro–Wilk tests. The data of CFU and gene expression were classified as non-normal and were transformed into exp(x) and log(x), respectively, to agree with the parametric statistics assumptions. Severity index data were analyzed by the non-parametric statistic test of Kruskal–Wallis. The data of plant height and dried mass, and *AREB2* expression, were classified as normal and were not transformed. The statistical analysis of all data used the R-software package [70,71]. Transformed and normal data were analyzed by analysis of variance (ANOVA) using the completely randomized design to identify significant differences among the treatments. Mean values were compared using Tukey’s and Duncan´s multiple comparison tests (*p <* 0.05). The trait mean values were normalized as median-centered Z-scores and used for a hierarchical clustering based on the Euclidean distance and the Ward’s linkage method. Heatmap used the heatmap.2 function available through the R package “gplots” [72].

## 4. Conclusions

The bicontrol of *Pst* needs a rigorous selection of the appropriate biological control agent. In the case of a COR-deficient *Pst* strain, it appears not to be a major problem. However, this task becomes compulsory when the pathogen is a COR-producing strain. This phytotoxin manipulates plant physiology, through the upregulation of SA- and JA-responsive genes, to such a degree that plant systemic defenses induced by *Trichoderma* may be unable to exert an effective protection against DC3000. However, in either case, *Trichoderma* is a vast reservoir of biocontrol agents and a strain-by-strain search is desirable, especially in difficult cases such as the induction of systemic defenses against insidious pathogens that like to produce COR. The present study gives some idea of how *Trichoderma* and *Pst* interact in tomato plants at a given time. Further time-course phytohormone networking gene expression studies accompanied by the measure of phytohormones such as SA, JA, ET, and ABA are deemed necessary to draw a complete picture of *Trichoderma*-induced defenses priming against *Pst*. A better understanding of when and how *Trichoderma* and *Pst* are applied and what informs the systemic defense process will allow us to progress in the development of eco-friendly plant protection products against this pathogen. 

## Figures and Tables

**Table 1:** Bacterial populations (BPs) and disease severity (DS) of *P. syringae* DC3000 and DC3118 strains in dip-inoculated tomato plants pre-treated with three *Trichoderma* species at 72 hpi of both bacterial strains.

Treatment	DC3000		DC3118	
	BP	DS ^b^	BP	DS
Control ^a^	6.24 b * A **	3 a A	5.80 a B	2 a B
T6	6.19 b A (0.8%) ***	3 a A	2.05 c B (64.7%)	1 a B
T25	5.65 c A (9.4%)	3 a A	2.11 c B (63.6%)	1 a B
T34	6.48 a A (−3.8%)	3 a A	4.80 b B (17.3%)	1 a B

For all control treatments (plants untreated with *P. syringae* (*Pst*)) colony-forming units (CFU) and DS values were zero. * Different lowercase letters, for comparisons among columns (show the effect among *Trichoderma* strains (T6 = *T. parareesei*, T25 = *T. asperellum,* and T34 = *T. harzianum*) for each *Pst* strain), indicate that the mean values are significantly different under Tukey’s test (*p* < 0.05). ** Different uppercase letters, for comparisons among rows (showing the effect of each *Trichoderma* treatment against both *Pst* strains), indicate that the mean values are significantly different under Tukey’s test (*p* < 0.05). ^a^ Four leaflets per plant and four biological replicates (*n* = 4) per treatment were considered for this study. The data represent the average of four replicates as log_10_ CFU/cm^2^ leaf for bacterial populations or as the severity index (range of 0 to 3) for the disease severity. *** Value in parentheses indicates the percentage of reduction in bacterial populations of DC3000 and DC3118 in relation to their control. For analysis of variances (ANOVA), the data of CFU were transformed into exp(x). ^b^ A non-parametric Kruskal–Wallis test was performed.

**Table 2:** Dry biomass and height in 4-week-old tomato plants at 72 hpi of *Pseudomonas syringae* pv. *tomato* under three *Trichoderma* treatments.

Treatment	Dry Biomass (g/Plant) ^a^	Height (cm)
Control	0.50 a *	13.65 a
T6	0.44 a	16.42 a
T25	0.41 a	14.61 a
T34	0.45 a	15.92 a

^a^ Data represent the average of twelve biological replicates (*n* = 12). Plants from each *Trichoderma* strain treatment (T6 = *T. parareesei*, T25 = *T. asperellum*, and T34 = *T. harzianum*), with and without *Pst* inoculation, were considered as a biological replicate. * Values in each column followed by the same letter do not differ significantly (Tukey’s test at *p* < 0.05).

**Table 3:** PAL activity in 4-week-old tomato plants 72 h post-*Pseudomonas syringae* pv. *tomato* inoculations (DC3000 and DC3118) under three *Trichoderma* treatments (T6 = *T. parareesei*, T25 = *T. asperellum*, and T34 = *T. harzianum*).

Treatment	Control	DC3000	DC3118
Control	173.55 abc *	257.89 c	177.35 abc
T6	115.07 a	209.59 bc	197.28 abc
T25	125.17 ab	188.34 abc	136.95 ab
T34	160.97 abc	213.86 bc	259.16 c

Data represent the average of four replicates (as U/g of fresh tissue). * Values in each column followed by the same letter do not differ significantly (Duncan’s test at *p* < 0.05).

## Supplementary Materials

The following are available online at https://www.mdpi.com/2223-7747/9/5/626/s1, Figure S1: Drawing scheme of tomato leaflets collected for counting bacterial population and for RNA extractions in this study, Table S1: List of specific primer pairs used in this study, Table S2: Effect of *Pst* and *Trichoderma* treatments on gene expression.
