# Supplementary material for: Differential Response of Tomato Plants to the Application of Three Trichoderma Species When Evaluating the Control of Pseudomonas syringae Populations
Source: Plants (Basel). 2020 May 14;9(5):626. doi: 10.3390/plants9050626 (PMC7285377; doi:10.3390/plants9050626)
Supplement: Supplementary file 1 [file plants-09-00626-s001.pdf]

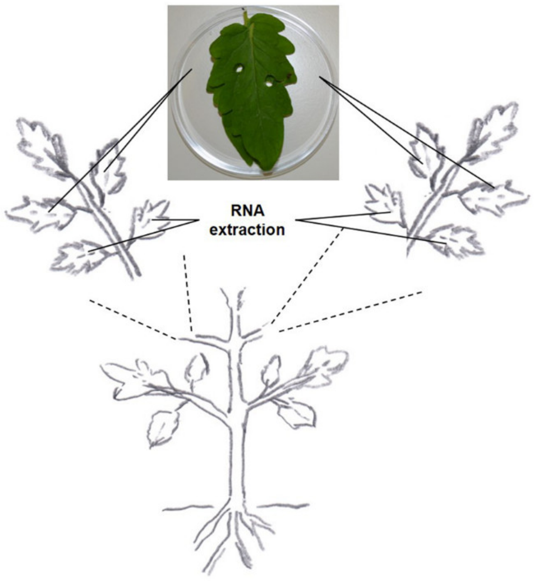

Figure S1. Drawing scheme of tomato leaflets collected for counting bacterial population and for RNA extractions in this study.

Table S1. List of specific primer pairs used in this study.

Gene	Plant signaling pathway	Forward (5'-3') / Reverse (5'-3')	Slope	Efficiency (%)	Accession Number	Reference
ICS1 (Isochorismate synthase)	Shikimate pathway for SA synthesis	GTTCCTCTCCAAGAAATGTCC / TCCTTCAAGCTCATCAAACTC	-3.32	100.12	XM_019214148	[45]
PAL5 (Phenylalanine ammonia lyase)	Phenylpropanoid pathway for SA biosynthesis	ACGGGTTGCCATCTAATCTG / TGGCTGAAATTAAGCCCAAG	-2.92	119.97	M83314	This study
PR1b1 (Pathogenesis-related (PR))	Salicylic acid (SA) response	GCACTAAACCTAAAGAAAAATGGG / AAGTTGGCATCCCAAGACATA	-3.17	106.94	Y08804	[37, 73]
TomLoxC (Lipoxygenase)	Jasmonic acid biosynthesis	TCCGGCAACACCGTTTACTC / GTCAATGGCCGGAAAATGTG	-3.44	95.28	U37839	[37]
MYC2 (Transcription factor MYC2)	Jasmonates response	CTGAAAAGAAGCCGAGGAAGC / GCATCTCCAAGAAGTGATGCC	-3.29	101.19	NM_001301178	[5]
PINII (Wound-induced	Jasmonates response	GGCCAAATGCTTGCACCTTT	-3.21	104.69	K03291	[37, 73]

proteinase inhibitor)		CGTGGTACATCCGGTGGGATA				
ERF-A2 (*ERF1*) (Ethylene response factor)	Ethylene response	AAGTGGCTCGCCTAAGAGGA<br>TAACATTTGGTCCCCGGCTC	-3.21	105.09	NM_001329459	[74]
AREB2 (ABA-responsive element binding protein 2)	Abscisic acid (ABA) response	GGTATCCCTGATCCAGCACTC<br>CACATAAGGAACTGGAGATACAG	-3.63	88.40	XM_026030430	[46]
LERBOH1 (NADPH oxidase)	Reactive oxygen species (ROS) biosynthesis	GTCAGGCTTCTACAGAAAAC<br>GTTGATTACAGTAGCCGGTTC	-3.12	109.08	AF088276	[75]
Actin-7-like	---	CACCACTGCTGAACGGGAA<br>GGAGCTGCTCCTGGCAGTTT	-3.30	101.06	NM_001321306	[37, 73]

Table S2. Effect of *Pst* and *Trichoderma* treatments on gene expression.

**Gene**	**Treatment**	**Control**		**DC3000**		**DC3118**		**P x T*** **
ICS1	**Control**	**0.83**	a* C**	18.93	a A	3.00	a B	$P < 0.05$
	**T6**	0.74	a C	5.76	b A	1.34	b B	
	**T25**	1.00	a C	15.92	a A	2.42	a B	
	**T34**	0.79	a C	6.60	b A	2.71	a B	
PAL5	**Control**	1.77	ab C	56.09	a A	8.46	bc B	$P < 0.05$
	**T6**	0.69	b C	37.84	a A	4.25	c B	
	**T25**	2.90	a B	27.98	a A	16.50	ab A	
	**T34**	2.64	a B	36.46	a A	32.39	a A	
PR1b1	**Control**	0.80	a B	3.47	b A	3.35	a A	$P < 0.05$
	**T6**	1.17	a C	16.78	a A	3.87	a B	
	**T25**	1.23	a B	2.21	b B	4.77	a A	
	**T34**	0.75	a B	4.91	b A	2.87	a AB	
TomLoxC	**Control**	0.73	a A	0.73	ab A	0.44	a A	$P < 0.05$
	**T6**	0.28	b B	0.70	b A	0.15	b B	
	**T25**	1.06	a A	0.93	ab A	0.72	a A	
	**T34**	1.31	a A	1.34	a A	0.63	a B	
MYC2	**Control**	0.90	a B	6.23	a A	0.91	a B	$P < 0.05$
	**T6**	0.88	a B	1.68	c A	0.51	b C	
	**T25**	0.98	a B	1.96	bc A	0.47	b C	
	**T34**	0.97	a B	2.84	b A	0.98	a B	
PINII	**Control**	0.86	a C	190.73	a A	3.40	a B	$P < 0.05$
	**T6**	0.75	a B	44.80	b A	0.74	b B	
	**T25**	0.58	a C	169.08	a A	1.80	a B	
	**T34**	1.13	a B	96.54	ab A	2.19	a B	
ERF-A2	**Control**	0.90	b B	5.45	b A	3.28	b A	$P < 0.05$
	**T6**	2.30	a B	14.50	a A	3.40	b B	
	**T25**	2.46	a B	5.51	b A	3.24	b B	

	**T34**	1.90	a C	21.73	a A	6.18	a B	
AREB2	**Control**	0.94	a B	4.03	a A	0.76	a B	*P* < 0.05
	**T6**	0.33	b B	2.17	b A	0.58	a B	
	**T25**	0.39	b B	1.90	bc A	0.49	a B	
	**T34**	0.36	b B	1.65	c A	0.65	a B	
LERBOH1	**Control**	0.92	a B	1.88	a A	1.00	a B	*P* > 0.05
	**T6**	1.24	a B	3.62	a A	0.78	a B	
	**T25**	0.96	a B	4.91	a A	1.16	a B	
	**T34**	0.64	a B	2.95	a A	0.76	a B	

Data analysis was performed for each individual gene. *Different lowercase letters, for comparisons among columns (showing the effect among *Trichoderma* strains (T6 = *T. parareesei*, T25 = *T. asperellum*, and T34 = *T. harzianum*) for each *Pst* strain and control plants), indicate that the mean values are significantly different under Tukey's test ($p < 0.05$). **Different uppercase letters, for comparisons among rows (showing the effect of each *Trichoderma* treatment against both *Pst* strains and control plants), indicate that the mean values are significantly different under Tukey's test ($p < 0.05$). The data represents the average of four replicates as the relative quantity (RQ, $2^{-\Delta\Delta Ct}$) of target genes compared to those of their basal condition (control plants untreated neither with *Trichoderma* nor with *Pst*). The expression values of each gene were normalized to the quantity of actin gene used as the endogenous gene. For statistical analysis, the data were transformed into log (x). ***Two-way analysis of variance (ANOVA) for interaction between factors (P = *Pst* and T = *Trichoderma* strains) (Tukey's test, $p < 0.05$).
